# An overview of online resources for intra-species detection of gene duplications

**DOI:** 10.3389/fgene.2022.1012788

**Published:** 2022-10-13

**Authors:** Xi Zhang, David Roy Smith

**Affiliations:** ^1^ Department of Biochemistry and Molecular Biology, Dalhousie University, Halifax, NS, Canada; ^2^ Institute for Comparative Genomics, Dalhousie University, Halifax, NS, Canada; ^3^ Department of Biology, Western University, London, ON, Canada

**Keywords:** comparative genomics, genome duplication, genome evolution, gene duplication, paralogous genes

## Abstract

Gene duplication plays an important role in evolutionary mechanism, which can act as a new source of genetic material in genome evolution. However, detecting duplicate genes from genomic data can be challenging. Various bioinformatics resources have been developed to identify duplicate genes from single and/or multiple species. Here, we summarize the metrics used to measure sequence identity among gene duplicates within species, compare several computational approaches that have been used to predict gene duplicates, and review recent advancements of a Basic Local Alignment Search Tool (BLAST)-based web tool and database, allowing future researchers to easily identify intra-species gene duplications. This article is a quick reference guide for research tools used for detecting gene duplicates.

## Introduction

Gene duplication can generate new genetic functions, a phenomenon which has been widely evidenced across the eukaryotic Tree of Life (Conant and Wolfe, 2008). There exist various models and mechanisms to explain the formation and retention of duplicated genes within genomes (Koonin, 2005; Innan and Kondrashov, 2010). For example, neutral processes can contribute to the evolution of gene duplication *via* genetic drift (Lynch, 2007; Brunet and Doolittle, 2018). Various adaptive hypotheses are available to explain how duplicate genes can be retained within species, such as the gene dosage hypothesis (Qian and Zhang, 2008) and the “escape from adaptive conflict” model (Des Marais and Rausher, 2008). There are five broad classes of mechanisms for generating gene duplicates, including whole-genome duplication (WGD) events, tandem duplications, transposon-mediated duplications, segmental duplications (also known as highly homologous sequence elements), and retroduplications, resulting from the “copy and paste” mechanism during reverse transcription (Panchy et al., 2016). In some instances, environmental conditions can impact the rate of fixation/loss of gene duplicates. For example, studies were carried out on the retention of duplicated genes involved in stress response, sensory functions, transport, and/or metabolism given specific environmental conditions (Kondrashov, 2012). Likewise, the yeast genomes *Saccharomyces cerevisiae* and *Schizosaccharomyces pombe* were explored to evidence gene duplication in organismal adaptation (Qian and Zhang, 2014). A large-scale genomic analysis of land plants was carried out to support gene duplication assisting the evolution of novel functions, such as the production of specific floral structures and disease resistance (Panchy et al., 2016). Regarding algae, it was discovered that gene dosage might play a role in the survival of the Antarctic green alga *Chlamydomonas* sp. UWO241 (renamed *Chlamydomonas priscuii*) *via* the retention of highly similar duplicate genes (HSDs) (Cvetkovska et al., 2018; Zhang et al., 2021a; Stahl-Rommel et al., 2022).

Multidomain protein structures, functional redundancy, and/or extensive small-scale duplication events are some of the major challenges in detecting gene duplicates (Li et al., 2001; Prince and Pickett, 2002; Li et al., 2003b). Moreover, when trying to identify duplicate genes within or across species it is often difficult to distinguish between orthologs vs. paralogs. The latter are homologous genes descended from a common ancestor via duplication events, while the former are homologs derived by speciation events (Lallemand et al., 2020). When identifying homologous genes within species, it is common practice to identify paralogs using similarity assessment metrics. When exploring homologous genes across multiple species, it becomes more challenging to differentiate paralogs and orthologs, especially among more distantly related species. However, there are some publicly available genome databases providing the classification and identification of paralogs and orthologs, such as NCBI (Pruitt et al., 2005) and Ensembl (Birney et al., 2004; Howe et al., 2021). The former allows users to select and compare gene orthologs in closely related species, while the latter allows researchers to analyze the submitted sequences in a tree-based pipeline (https://useast.ensembl.org/info/genome/compara/homology_method.html) where the gene trees are reconciled against species trees to distinguish duplication and speciation events (i.e., paralogues and orthologues). Besides, there are various available methods for identifying orthologous genes by building orthologous groups in multispecies (Kuzniar et al., 2008; Altenhoff and Dessimoz, 2012). For example, tree-based methods usually recognize groups of genes based on the inferred types of relationship ahead of building a phylogenetic tree, such as TreeFam (Schreiber et al., 2014) and PhylomeDB (Huerta-Cepas et al., 2014); however, the multispecies, graph-based methods need to form the homology graph first and then build sets of genes dependent on the types of suggested relationships, such as OrthoMCL (Li et al., 2003a) and OrthoFinder (Emms and Kelly, 2019).

Here, we focus on gene duplication detection resources for intra-species analyses and review recent advancements in this area. We first summarize the metrics used to measure the similarity of gene duplicates within species, then compare several computational approaches that have been used to predict and collect gene duplicates within a particular genome. In addition, we review the recent development of a Basic Local Alignment Search Tool (BLAST)-based web tool (HSDFinder) (Zhang et al., 2021b; Zhang et al., 2021c) and database (HSDatabase) (Zhang et al., 2022). Using these two bioinformatics resources, a comparative platform can be built to understand the role of gene duplication in genome evolution.

## Metrics for measuring sequence similarity of gene duplicates

Measuring duplicated genes within species typically involves the gene structure method and/or sequence similarity method. For example, three metrics are usually applied to evaluate the sequence similarity of the paralogous relationships in genes, such as aligned length, sequence identity and E-value (Lallemand et al., 2020). Other kinds of metrics are also available, but they are not necessarily as straightforward to measure (e.g., bit-score). Sequence similarity and alignment length of genes can be rapidly quantified by many tools, including DIAMOND (Buchfink et al., 2015) and BLAST (Kent, 2002). When identifying gene duplicates, the amino acid sequence is typically preferred over the nucleotide sequence as the former is more evolutionarily conserved providing more reliable sequence alignments as compared to DNA sequences. This is also why many gene duplication detection tools have the input files running from BLASTP or BLASTX (Kent, 2002). Furthermore, the timescale of the gene duplicates can greatly impact the selection of different metrics in the alignment software. Filtering recent gene duplicates usually requires more restrictive thresholds and vice versa. The metrics used to define the paralogs in a BLAST all-against-all amino acids sequence search usually include a smaller E-value cut-off (e.g., ≤ 1e-5), a higher identity score (e.g., ≥ 30%), and a longer aligned length (e.g., ≥ 150 amino acids) (Sander and Schneider, 1991; Maere et al., 2005; Panchy et al., 2016).

To overcome the limitations of similarity-based assessments, efforts have been made in developing various similarity-based metrics. For example, the homology-derived secondary structures of proteins (HSSP) method (Sander and Schneider, 1991) creates a formula to help researchers quantify genetic paralogous relationships (Rost, 1999; Li et al., 2001). Many databases have been developed to collect the conserved domains and pathways, which can be used to infer gene similarity (Lallemand et al., 2020), such as Pfam database (El-Gebali et al., 2019), InterPro pattern (Mitchell et al., 2019), and KEGG pathway (Kanehisa and Goto, 2000). But it should be noted that the quality of the genome assembly and annotation can play a key role in the accuracy of gene similarity assessement analyses. For example, ‘duplicate’ contigs from different haplotypes can remain in the final genome assemblies (especially for heterozygous genomes), potentially leading to false detection of gene duplicates; although this has been improved considerably with long-read sequencing technologies. In Table 1, various species were assessed for gene similarity showing that the observed numbers of gene duplicates can be distinct with each given threshold and assembled genome.

**TABLE 1:** Estimation of the amount of duplicated genes in different species. Adapted from (Lallemand et al., 2020) under the creative commons attribution license.

Species	No. of median gene count	No. of estimated gene copies	Percentage of estimated gene copies	Duplicated gene types	References
*Arabidopsis thaliana*	25,557	11,937	46.7	Not specified, all paralogous pairs were searched^a^	Blanc and Wolfe, (2004)
	22,810	21,622	94.8	WGD, tandem, proximal, DNA based transposed, retrotransposed, and dispersed duplications^b^	Wang et al. (2011), Lee et al. (2012)
	27,558	12,761	46.3	Not specified, genes families were obtained^c^	Maere et al. (2005)
	27,560	14,225	51.6	All paralogous pairs were searched^d^	Zhang et al. (2022)
*Homo sapiens (human)*	19,727	12,981	65.8	Gene families (tandem duplications searched among families)^e^	Shoja and Zhang, (2006)
	20,415	15,569	76.3	WGD and SSD^f^	Singh et al. (2015)
	22,447	11,740	∼52.3	WGD and SSD^g^	Acharya and Ghosh, (2016)
	19,531	6,352	32.5	All paralogous pairs were searched^d^	Zhang et al. (2022)
*Mus musculus (mouse)*	21,305	14,043	65.9	Gene families (tandem duplications searched for among families)^f^	Singh et al. (2015)
	27,736	16,091	∼58.0	Ensembl family database and genes >300 nt. Tandem duplications were then searched for among families^h^	Pan and Zhang, (2008)
	30,736	8,855	28.8	All paralogous pairs were searched^d^	Zhang et al. (2022)
*Rattus norvegicus (rat)*	18,468	12,466	67.5	Gene families (tandem duplications searched for among families)^e^	Singh et al. (2015)
	27,194	16,446	∼60.5	Gene families (tandem duplications searched for among families)^h^	Pan and Zhang, (2008)
	22.219	8,757	39.4	All paralogous pairs were searched^d^	Zhang et al. (2022)
*Oryza sativa (rice)*	18,562	9,149	49.3	Not specified, all paralogous pairs were searched^i^	Blanc and Wolfe, (2004)
	27,910	21,461	76.9	WGD, tandem, proximal, DNA based transposed, retrotransposed, and dispersed duplications^b^	Wang et al. (2011); Lee et al. (2012)
	28,735	14,704	51.2	All paralogous pairs were searched^d^	Zhang et al. (2022)
*Zea mays (maize)*	∼62,000	∼43,000	∼69.0	All paralogous pairs were searched^j^	Panchy et al. (2016)
	34,328	22,499	65.5	All paralogous pairs were searched^d^	Zhang et al. (2022)

a All-against-all nucleotide sequence similarity searches using BLASTN, among the transcribed sequences. Sequences aligned over >300 bp and showing at least 40% identity were defined as pairs of paralogs.

b All-against-all protein sequence similarity search using BLASTP (top five non-self protein matches with E-value of 1e-10 were considered). Genes without hits that met a threshold of E-value 1e-10 were deemed singletons. Single gene duplications were derived by excluding pairs of WGD, duplicates from the population of gene duplications. Tandem duplications were defined as being adjacent to each other on the same chromosome. Proximal duplications were defined as non-tandem genes on the same chromosome with no more than 20 annotated genes between each other. Single gene transposed-duplications were searched for from the remaining single gene duplications using syntenic blocks within and between 10 species to determine the ancestral locus. If the parental copy had more than two exons and the transposed copy was intronless, the pair of duplicates was classified as coming from a retrotransposition. Other cases of single gene-transposed duplications were classified as DNA-based transpositions. Dispersed duplications corresponded to the remaining duplications not classified as WGD, tandem, proximal, or transposed duplications (Lee et al., 2012; Wang et al., 2011).

c All-against-all protein sequence similarity search using BLASTP (E-value cutoff of 1e-10). Sequences alignable over a length of 150 amino acids with an identity of 30% were defined as paralogs. Gene families were built through single-linkage clustering.

d A combination of thresholds was used to acquire a larger dataset of HSD candidates (Zhang et al., 2022). All-against-all protein sequence similarity search using BLASTP (E-value cutoff of ≤1e−10) filtered via the criteria with in certain amino acid length differences and larger than certain amino acid pairwise identities. HSD candidates were added one after another at different similarity assessment metrics (i.e., HSDs identified at more relaxed thresholds were treated more strictly than those found using more conservative thresholds). For example, HSDs identified at a threshold of 90%_30aa were added on to those identified at a threshold of 90%_10aa (denoted as “90%_30aa+90%_10aa”); any redundant HSD candidates picked out at this combination threshold were removed if the more relaxed threshold (i.e., 90%_30aa) had the identical genes or contained the same gene copies from the stricter cutoff (i.e., 90%_10aa). Moreover, any HSD candidates pinpointed at the combination threshold (90%_30aa+90%_10aa) were removed if the minimum gene copy length was less than half of the maximum gene copy length for each HSD, or if HSD candidates had gene copies with incomplete conserved domains (i.e., different number of Pfam domains). After filtering the combination threshold at (90%_30aa+90%_10aa), a more relaxed threshold 90%_50aa was added on [i.e., 90%_50aa+(90%_30aa+90%_10aa)] and then carried out the same HSD candidate removal/filtering process. To minimize the redundancy and to acquire a larger dataset of HSD candidates, each selected species was proceeded with the following combination of thresholds: E + {D + [C + (B + A)]}. A = 90%_100aa+{90%_70aa+[90%_50aa+(90%_30aa+90%_10aa)]}; B = 80%_100aa+{80%_70aa+[80%_50aa+(80%_30aa+80%_10aa)]}; C = 70%_100aa+{70%_70aa+[70%_50aa+(70%_30aa+70%_10aa)]}; D = 60%_100aa+{60%_70aa+[60%_50aa+(60%_30aa+60%_10aa)]}; E = 50%_100aa+{50%_70aa+[50%_50aa+(50%_30aa+50%_10aa)]}.

e All-against-all protein sequence similarity search using BLASTP, with the BLOSUM62 matrix and the SEG filter, TribeMCL, with the default parameters. Tandem duplications were then searched for among families.

f Pooling of different datasets from Singh et al. (2015) and all-against-all protein sequence similarity search using BLASTP. WGD refers to whole genome duplication, SSD refers to small-scale duplication.

g Ensembl version 77, >50% sequence identity, and high confidence for paralogy.

h Ensembl family database and genes >300 nt. Tandem duplications were then searched for among families.

i All-against-all nucleotide sequence similarity searches using BLASTN, were done among the transcribed sequences. Sequences aligned over >300 bp and showing at least 40% identity were defined as pairs of paralogs.

j A gene is regarded as duplicated if it is significantly similar to another gene in a BLAST search (identity ≥30%, aligned region ≥150 amino acids, E-value cutoff of ≤1e−5).

## Bioinformatics approaches to identify gene duplicates

Researchers have been studying gene duplication for years, which has led to the development of various bioinformatics databases and tools for within and/or among genomes/species analyses (Lallemand et al., 2020). It is important to know how these tools function in order to choose the correct one for studying gene duplicates. There are a few factors for future researchers to consider, such as genome structure (e.g., diploid or haploid; plant or animal; eukaryotic or prokaryotic), the specific questions being asked (e.g., WGD genes or retrogenes; tandem duplicates or segmental duplicates), and the bioinformatics skills needed (e.g., command line environment or graphical user interface). Also, as noted above, the challenges associated with distinguishing orthologs from paralogs increase when exploring homologous genes between distant species. But there are still some tools available, such as the graph-based duplication prediction software OrthoMCL (Li et al., 2003a), which has a built-in Markovian Cluster algorithm, and the popular orthologous protein-coding genes database OrthoDB (Zdobnov et al., 2017). Besides, researchers developed an efficient and simple-to-use tool OrthoFinder (Emms and Kelly, 2015; Emms and Kelly, 2019) aimed at detecting the relationship of orthologous groups between/among species, especially one-to-many and many-to-many relationships between orthologues. This allows unique orthologous genes can to be collected using a reciprocal best hits (RBH) approach, which gets more complex as the number of gene duplication events increases. OrthoFinder can detect these relationships and provide comprehensive statistics for comparative genomic analyses via protein sequence files (one per species) in FASTA format. Despite the convenience of these tools, there is still an increasing need for bioinformatics tools and databases for studying specific types of gene duplications within a particular genome.

There are many web tools and databases devoted to within-species gene duplication analysis, some of which are no longer maintained. Table 2 presents the different types of algorithms used in these software/databases with a focus on those that are recently developed and/or actively maintained. For example, co-localized gene duplicates were collected from nine species in an early developed database named Duplicated Gene Database (DGD); however, it appears that no new species have been added since 2012 (Ouedraogo et al., 2012). Two genes are treated as co-localized relationship in the DGD only when they fit in the 100 gene window of all-against-all BLAST results and meet the following criteria (*I’ = I x Min(n*_*1*_ */L*_*1*_ *,n*_*2*_ */L*_*2*_ *)*; *I’* ≥30% if *L* ≥ 150 amino acids; *I* is the sequence identity, *L*_*i*_ is the length of sequence, *n*_*i*_ is the number of amino acids in the aligned region) and formula (*I ≥ 0.01n+4.8L* ^*−0.32(1+exp(−L/1000))*^) (Li et al., 2001). The database RetrogeneDB provides detailed data on retrogene duplicates, which must have at least 50% amino acid identity and coverage to the location from which they initially arose from, and be at least 150 bp long (Kabza et al., 2014; Rosikiewicz et al., 2017). PTGBase is built as an integrated database focusing on tandemly duplicated genes in plants; the tandem duplicates were collected by looking at if two or more genes from the same orthologous group are next to each other in the target genome (Yu et al., 2015). Similarly, gene and genome duplication of representative plant genomes were collected in the Plant Genome Duplication Database (PGDD) (Lee et al., 2012; Lee et al., 2017). More recently, Wang and colleagues developed a duplication events detection pipeline, called DupGen_finder, which has the built-in algorithm of MCScanX (Wang et al., 2013) and can identify duplicates of different type, such as tandem, whole-genome, transposed, proximal, or dispersed duplications (Wang et al., 2011; Qiao et al., 2019).

**TABLE 2:** Summary of the characteristics of different existing tools for identifying gene duplicates.

Name	Input	Output Text	Output Plots	Main algorithm	Specificities	Other information	Resource links	Programming languages	Interface	References
Duplicated Gene Database (DGD)	Protein sequences and gene annotations data from Ensembl Flicek et al. (2013)	Tabulated txt	None	DGD defines groups of duplicated using Rost’s Blast Rost (1999) parameters analysis	Using a maximum genomic distance of 2.5 MB between two putative duplicated genes	Not updated any new species since 2012	Web: http://dgd.genouest.org	No requirements	Web user interface	Ouedraogo et al. (2012)
Plant Genome Duplication Database (PGDD)	Coding DNA sequences, protein sequences and general feature format (GFF) file	Tabulated txt	Graphical visualization	Providing genome alignments from a single resource based on uniform standards that have been validated	Providing synteny information in terms of colinearity between chromosomes Wang et al. (2013)	The web link from the publication is no longer working	Web: http://chibba.agtec.uga.edu/duplication/	No requirements	Web user interface	(Lee et al., 2012; Lee et al., 2017)
DupGen_finder; PlantDGD	Pre-computed BLAST results (-outfmt 6) and gene location information (GFF file)	Tabulated txt	None	Each duplicate gene was assigned to a unique mode after all of the duplicated gene pairs were classified into different gene duplication types	Including duplicate genes derived from whole-genome, tandem, proximal, transposed, and dispersed duplication that was identified using uniform standards	MCScanX algorithm Wang et al. (2013) was incorporated in this pipeline	GitHub: https://github.com/qiao-xin/DupGen_finder; Web: http://pdgd.njau.edu.cn:8080	Perl	Web user interface and command line	(Wang et al., 2011; Qiao et al., 2019)
PTGBase	Coding DNA sequence file, protein sequence file and general feature format file	Tabulated txt	None	Using in-house scripts to look at phylogenetic relationship, location of gene models, and tandem duplicated arrays	Functional annotation of tandem duplicated genes including InterPro and Gene Ontology (GO)	The web link from the publication seems not working at the day of writing (20 June 2022)	Web: http://ocri-genomics.org/PTGBase/	No requirements	Web user interface	Yu et al. (2015)
RetrogeneDB	All sequences of all species were downloaded from Ensembl 73 Flicek et al. (2013) and Ensembl Plants 30 Kersey et al. (2016)	Tabulated txt	Graphical visualization	Using the LAST program Kielbasa et al. (2011) by the translated protein sequence alignment to the hard-masked reference genome sequence	Genes that contain a reverse transcriptase domain were excluded from the set.	The database has updated to a secondary version	Web: http://yeti.amu.edu.pl/retrogenedb; http://rhesus.amu.edu.pl/retrogenedb	No requirements	Web user interface	(Kabza et al., 2014; Rosikiewicz et al., 2017)
HSDFinder	BLASTP output of protein sequence and associated InterProScan and KEGG annotation file	Tabulated txt	Graphical visualization (heatmap plot)	Collecting by all-against-all BLAST and grouping by a simple transitive link between remaining genes	Highly relied on the similarity metrics provided	Can identify all pairs of gene duplicates only if they are satisfied with certain criteria	GitHub: https://github.com/zx0223winner/HSDFinder; Web: http://hsdfinder.com	Python, Bash	Web user interface and command line	(Zhang et al., 2021b; Zhang et al., 2021c)
HSDatabase	A list of species name, gene name and the respective HSDFinder results	Tabulated txt	Graphical visualization (Genome browser and sequence alignment)	A series of combination thresholds were applied to collect and curate HSDs from a diversity of species	To find paralogs that are highly similar in sequence and thus likely carry out the same function	Can be used on its own for comparative analyses of gene duplicates or in conjunction with HSDFinder	Web: http://hsdfinder.com/database/	No requirements	Web user interface and command line	Zhang et al. (2022)

## Recent advancement of a BLAST-based web tool and database

The psychrophilic, Antarctic green alga *Chlamydomonas priscuii* was recently shown to contain hundreds of highly similar duplicate genes, which may be helping this species survive extreme conditions via a gene dosage effect (Cvetkovska et al., 2018; Zhang et al., 2021a; Stahl-Rommel et al., 2022). A novel HSD detection tool, called HSDFinder, was developed for analyzing gene duplicates in *C. priscuii* (Zhang et al., 2021b; Zhang et al., 2021c). This tool has now been applied to many other eukaryotic genomes, the results of which are available in a online database called HSDatabase, housing 117,864 HSDs arising from 40 eukaryotic species (Zhang et al., 2022). HSDatabase contains an assortment of user-friendly features allowing users to glean important information on HSDs, including alignment length and percentage identify, and it provides external links to NCBI’s genome browser, Pfam protein domains, and KEGG pathways. Furthermore, HSDatabase has a built-in BLAST tool for users to search genes of interest.

With this newly developed tool, BLAST all-against-all amino acid sequences can be used as the input file for the web server - HSDFinder (Zhang et al., 2021b) to furtherly explore sequence similarity. With a user-friendly interface, amino acid length variance and sequence identity can be conveniently submitted as similarity assessment metrics. By using these metrics, duplicate genes are grouped by a simple transitive link between remaining genes. There is an online heatmap option for users to compare intra-species gene duplicates under different thresholds. The KEGG pathway framework is used to categorize the detected duplicates in the heatmap.

A combination of thresholds (relaxed ones added onto stricter ones) was developed to acquire a larger dataset of HSD candidates in HSDatabase (Zhang et al., 2022). Also, any HSD candidates were screened out if the minimum length of gene copy was less than half of the maximum length of gene copy for every HSD group. Incomplete or unequal conserved protein family domains of HSD candidates will also result in the removal of the HSD group. But due to the limitation of this strategy, it should be noted that there are some large groups of HSD candidates in the database that likely diverged in function from one another. In the database, those putatively diverged HSD groups were labelled as “candidate HSDs” and a warning note was added that users should proceed with caution when working with these types of datasets.

## Concluding perspectives

There is no stand-alone software that can detect all types of gene duplicates within and across species. There are many factors that can influence the choice of tools being used for gene duplication detection. These include, for instance, the kinds of questions being asked and the genomes being analyzed as well as the bioinformatics skills of the user. For developers, a lot of features and statistics can be added to assist future researchers, such as the rates of synonymous and nonsynonymous substitutions (dN/dS rates) and differential expression levels in different gene duplicates. One of the big challenges moving forward is how to properly help users select an appropriate threshold for their given dataset/genome and provide them with the freedom to fine-tune specific metrics. In the future, it is likely that users will be aided by species-specific gene threshold values for gene duplication detection tools. With more and more genomes being sequenced and re-sequenced, gene duplicate data from highly polished model genomes will broaden our understanding of the role of gene duplication in genome evolution and adaptation to extreme environments.
